# Prevention of Chronic Rejection of Marginal Kidney Graft by Using a Hydrogen Gas-Containing Preservation Solution and Adequate Immunosuppression in a Miniature Pig Model

**DOI:** 10.3389/fimmu.2020.626295

**Published:** 2021-02-17

**Authors:** Kotaro Nishi, Satomi Iwai, Kazuki Tajima, Shozo Okano, Motoaki Sano, Eiji Kobayashi

**Affiliations:** ^1^Laboratory of Small Animal Surgery 2, School of Veterinary Medicine, Kitasato University, Towada, Japan; ^2^Laboratory of Small Animal Internal Medicine 2, School of Veterinary Medicine, Kitasato University, Towada, Japan; ^3^Department of Surgery, Keio University School of Medicine, Tokyo, Japan; ^4^Department of Cardiology, Keio University School of Medicine, Tokyo, Japan; ^5^Department of Organ Fabrication, Keio University School of Medicine, Tokyo, Japan

**Keywords:** kidney transplantation, hydrogen-containing organ preservation solution, chronic allograft nephropathy, chronic rejection, marginal donor, mature minipig model, multi-immunosuppressant

## Abstract

In clinical kidney transplantation, the marginal kidney donors are known to develop chronic allograft rejection more frequently than living kidney donors. In our previous study, we have reported that the hydrogen gas-containing organ preservation solution prevented the development of acute injuries in the kidney of the donor after cardiac death by using preclinical miniature pig model. In the present study, we verified the impact of hydrogen gas treatment in transplantation with the optimal immunosuppressive protocol based on human clinical setting by using the miniature pig model. Marginal kidney processed by hydrogen gas-containing preservation solution has been engrafted for long-term (longer than 100 days). A few cases showed chronic rejection reaction; however, most were found to be free of chronic rejection such as graft tissue fibrosis or renal vasculitis. We concluded that marginal kidney graft from donor after cardiac death is an acceptable model for chronic rejection and that if the transplantation is carried out using a strict immunosuppressive protocol, chronic rejection may be alleviated even with the marginal kidney.

## Introduction

The number of patients with end-stage chronic kidney disease is high worldwide; this is a crucial issue because of the substantial cost of medical treatment (1). A considerable number of patients with end-stage chronic kidney disease are awaiting kidney transplantation because of a chronic shortage of living donors. As a result, dialysis is required for these patients. Although the survival of grafts in living kidney transplantation has been greatly improved by the emergence of effective calcineurin inhibitors and antimetabolites (2, 3), it is critical to secure organs from a wide range of donors to solve the problem of living donor shortage. Moreover, effective utilization of marginal organs from donors after cardiac death (DCD) is required.

Marginal kidneys from DCD carry a high risk of short-term or long-term graft loss, depending on the donor’s individual circumstances (including age, sex, blood pressure, and medical history) (4). In particular, ischemia-reperfusion injury (IRI) due to prolongation of warm ischemic time in the transplanted kidney causes strong expression of cytokines and oxidants such as IL-1, IL-6, and TNF-α, resulting in primary nonfunction and delayed graft function (4–7). In addition, T cell- and antibody-mediated rejection of kidney tissues is factors that exacerbate acute rejection (8, 9). The presence of acute rejection may trigger and lead to chronic allograft rejection. Thus, IRI increases the risk of developing a chronic allograft rejection over the long-term post-transplantation period, and outcomes have not improved despite appropriate immunosuppressant therapy after surgery (3, 10). It is known not only in humans but also in animal models that ischemic kidney transplantation from DCD is less likely to survive long-term and undergo chronic rejection than transplantation from living donors (11, 12). It has been reported that chronic allograft rejection involves immunological factors such as HLA histocompatibility and acute rejection and non-immunological factors such as nephrotoxicity by calcineurin inhibitor and ischemia-reperfusion (13, 14). However, only about 60% of chronic graft damage can be explained by these risk factors (15). To date, several studies have been conducted on mechanical perfusion and the inclusion of oxygen and carbon monoxide in organ preservation fluids to reduce acute and chronic damage, some of which are used clinically (16–18). However, despite the benefits, chronic allograft nephropathy remains a highlight. Therefore, it is thought that the mechanism of chronic allograft rejection involves a combination of several of these factors.

Currently, there have been several studies that attempted to alleviate acute and chronic allograft rejection by improving IRI during transplantation at an early stage, using animal models such as rodents, rabbits, dogs, and pigs (1, 19). In particular, the rat model was used in several studies owing to its advantages of low cost, easy maintenance, and established techniques for blood vessel and ureteral anastomosis. Furthermore, in humans, chronic changes such as thickening of the blood vessel wall and atrophy of the kidney tubules develop over several years to decades, whereas in rat models, these results, as well as chronic tissue changes, can be observed within several months (20). Nevertheless, these rats are typically young and male, with mild ischemic injury and an observation period of <3 months, and does not show clinical manifestations of chronic allograft rejection. Furthermore, most rat strains used have been allogeneic transplants when the rat MHC antigen subtypes such as F344 and LEW were the same or were a combination of strains with low rejection (20–23). There have been few papers describing control of ischemic kidney transplantation using a combination of multiple immunosuppressants in a system with strong rejection, assuming actual clinical practice (13, 24, 25). In a rat study, allograft tolerance occurred by administering a single immunosuppressant for several weeks to several months after surgery. From the viewpoint of chronic allograft nephropathy, this also makes it difficult to extrapolate to human transplant recipients who are maintained using several immunosuppressants.

In a previous study, we reported that hydrogen-containing organ preservation solution for rinsing and preservation of grafts reduced acute phase IRI in kidneys of adult minipigs (26). The anti-inflammatory effects of hydrogen, such as inhibiting inflammatory cytokines, inducing HO-1, and activating NF-E2-related factor, have been reported in transplanted organs (27, 28). This solution has been shown to be effective in suppressing IRI in transplanted organs such as the heart, lung, and kidney (29–34). In the present study, we aimed to verify the impact of hydrogen gas treatment in the prevention of chronic rejection of marginal kidney graft with an optimal immunosuppressive protocol based on human clinical setting by using the miniature pig model.

## Materials and Methods

The pigs used in this study were micro-miniature pigs (Fujimicra Ltd., Shizuoka, Japan) with body weights not exceeding 30 kg even when mature, as previously described (26, 35, 36). We used four males and eight females, with mean age of 25.8 ± 5.5 months (range, 14.8–37.7 months; median, 25.4 months) and mean weight at 20.9 ± 2.4 kg (range, 17.4–26.8 kg; median, 20.3 kg). The pigs were randomly selected donors and recipients and had different swine leukocyte antigen (SLA) suitability. The pigs were housed in cages under temperature- and light-controlled conditions (12-h light/dark cycle) and were provided with food and water *ad libitum*. The breeding room was clean and maintained by washing twice daily. These experiments were conducted with the approval of the Research Council and Animal Care and Use Committee of Kitasato University (approval number, 18-133, 19-086). The animal experiments were performed according to the ARRIVE (Animal Research: Reporting of *In Vivo* Experiments) guidelines 2.0 as a guideline for animal experiments, and all necessary checklist criteria were met.

### Pre-Operative Examination and Treatment

Blood and urine tests were performed at screening before surgery to confirm absence of health-related abnormalities, and blood compatibility testing was completed to determine the matching donor and recipient settings in all cases. The recipients began receiving tacrolimus (0.15 mg/kg, PO, BID) and mycophenolate mofetil (500 mg/head, PO, BID) for the induction of immunosuppression 2 days before kidney transplantation.

### Preparation for Hydrogenation

The organ preservation solution used for rinsing and preservation of organs was extracellular-type trehalose-containing Kyoto (ETK), which has been shown to have a renal-preservation effect in previous studies (37). The hydrogen addition method to ETK was performed using a hydrogen storage alloy canister as described (26). The hydrogen was fed from a rubber stopper into a bag of ETK cooled to 4°C and mixed when the internal pressure of the container reached 0.06 ppm. The hydrogen concentration decreased slowly when stored at 4°C under normal pressure, and dissolved hydrogen concentration of 1 ppm or more could be secured for 4 h. Therefore, the pressure reduction was similar to that reported previously and the product was stored at 4°C (26). These operations were performed immediately before ischemic nephrectomy of the donor.

### Anesthesia Methods

The donor and the recipient at the time of transplantation were sedated by intramuscular administration of a mixed solution of medetomidine (20 µg/kg), midazolam (0.2 mg/kg), and butorphanol (0.2 mg/kg) to the gluteal muscle. After sufficient sedation was induced, mask inhalation was performed with isoflurane of 3.0%, and anesthesia was induced until the pharyngeal reflex disappeared. The 24-G catheter indwelling needle was inserted into the central ear vein, and lactated Ringer’s solution was started at 5 ml/kg/h. The infusion volume was changed appropriately by monitoring anesthesia during surgery. Then, a 5.5-Fr tracheal tube was introduced, and anesthesia was maintained with isoflurane 2.0%. An 8-Fr balloon catheter was placed in the bladder of the recipient to secure visuals of the surgical field and confirm urination during surgery. The catheter was removed after surgery. All pigs were administered with enrofloxacin [5 mg/kg, SC (subcutaneously), BID (bis in die/twice daily)] and cefazolin sodium hydrate (30 mg/kg, IV, BID) as antibiotics and buprenorphine (20 µg/kg, IV, BID) as an analgesic from the preoperative period.

### Kidney Transplantation Procedure

Two kidneys obtained from one donor were transplanted to two recipients, and both kidneys of the recipients were excised. The group using hydrogenated ETK was classified as H-DCD20 (n = 4) and the normal ETK group as NH-DCD20 (n = 3). The living kidney-transplanted individual (DCD 0, n = 1) was treated with the same protocol as the other groups except for ischemic manipulation.

**Figure 1:**
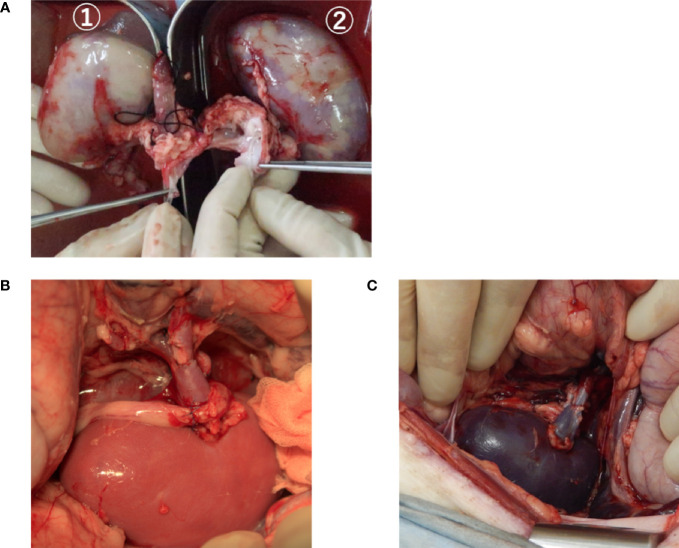
The kidneys during rinse and after reperfusion of blood. **(A)** A typical image showing the kidney being rinsed by the organ preservation solution. The kidney of ① is rinsed with a hydrogenated organ preservation solution. ② is rinsed with a non-hydrogenated organ preservation solution. The whole area of ① is flushed compared to ②. **(B, C)** The transplanted kidney immediately after reperfusion after rinsing and storage with an organ preservation solution. B, hydrogenated kidney; C, non-hydrogenated kidney.

The donor was anesthetized and held in a supine position. The chest and abdomen were shaved and disinfected. A midline incision was made in the abdomen with an electric scalpel. Blood flow to the aorta and posterior vena cava was blocked on the cranial and caudal sides of the kidney arteries and veins to induce kidney ischemic time for 20 min. An anticoagulant was not used in blocking the blood flow of the donor. Twenty minutes later, the abdominal aorta and posterior vena cava, left and right kidneys, and ureter were separated and excised. The excised kidney was immediately cannulated from the kidney artery and rinsed with cooling hydrogenated ETK or normal cooled ETK from a height of 1 m by natural dropping for 10 min (**Figure 1A**). Further, it was immersed in a cooled storage solution similar to the rinse liquid. Excessive soft tissue around the kidney hilum was trimmed in the preservation solution, and anastomosis of the blood vessel was formed. The kidneys were stored for 60 or 240 min in the same preservation solution as the rinse solution and allowed to stand until transplantation.

The recipient underwent anesthesia as per the donor protocol. A midline incision was made in the abdomen, and we incised the retroperitoneum to separate the kidney and renal artery and vein on the side to be transplanted. The vascular anastomosis method was determined by the confirmed shape of the blood vessel of the transplanted kidney. The blood flow of the abdominal aorta or renal artery, posterior vena cava, or renal vein was blocked with a vascular clamp, after which the recipient-kidney was removed, and then, the anastomosed blood vessel was formed. Heparin (1,000 units/head) was administered intravenously and was administered again 2 h after the first administration. The donor-kidneys were taken out from the preservation solution and placed in the abdominal cavity of the recipient after the preservation time of the donor-kidney was finished. Subsequently, the kidney artery was sutured for end-to-side or end-to-end anastomosis with 5–0 monofilament non-absorbable thread and the kidney vein with 6-0 monofilament non-absorbable thread. In the case of anastomosis to the abdominal aorta, the ischemic time of the recipient was set to within 30 min from clamping of the abdominal aorta. After the arterial anastomosis was competed, the blood vessel clamp was moved to the renal artery, and the blood flow in the abdominal aorta was restarted. Following kidney arteriovenous anastomosis, the blood flow block was released, and blood flow into the kidney and normal pulsation of the kidney artery were confirmed (**Figures 1B, C**). The ureter was anastomosed with 6-0 monofilament non-absorbable thread and the retroperitoneum was sutured. The abdominal wall was sutured using 1-0 and 3-0 synthetic multifilament absorbent threads. Furthermore, the skin was routinely sutured using 2-0 nylon thread. An access port for sampling blood and drug administration was installed in the jugular vein using a central vein (CV) catheter kit. The CV catheter in the surviving pig was removed 1 month after surgery.

### Perioperative Management and Long-Term Observation

The pigs receiving kidney transplantation were provided with food and water *ad libitum* from the first day after surgery. Antibiotics and analgesics were administered postoperatively, and tacrolimus [0.1 mg/kg/day, IV (intravenously)] was intravenously administered continuously until ingestion was observed if the animal had no appetite. Tacrolimus (0.15–0.6 mg/kg, p.o., BID), mycophenolate mofetil (250–500 mg/head, po, BID), and methylprednisolone (0.5–2 mg/kg, IV or PO, BID) were administered during the follow-up period. Tacrolimus and mycophenolate mofetil doses were set based on previous reports (38). Tacrolimus trough levels were measured routinely and the dose adjusted to maintain trough levels of 5–15 ng/ml. When a decrease in the trough level of tacrolimus was observed, the dose of tacrolimus was gradually increased to adjust the dose. In situations considered to represent acute rejection, the dose of prednisolone was increased for 3 days (2 mg/kg, BID) and gradually decreased over 1 week. As an analgesic, buprenorphine (20 µg/kg, IV, BID) was administered. As appropriate, famotidine (0.5 mg/kg, PO or IV BID) was orally or intravenously administered, as well as darbepoetin (30 μg/head, SC, once a week) and iron which was administered orally.

Blood sampling from the CV catheter was performed until the kidney function stabilized. Blood was collected every day until the first week after the operation and at 2 weeks, 1 month, 2 months, and 100 days postoperatively. We recorded the complete blood count (CBC), blood urea nitrogen (BUN), and creatinine levels. In addition, syndecan-1 levels were measured using plasma before surgery and 4 h after transplantation using an ELISA kit (MBS101321, MyBioSource, CA, USA), because the increase in syndecan-1 levels due to angiopathy occurs at approximately 2–4 h postoperatively, according to a previous report (39). Urine was collected at the same time as blood sampling, and the urine was evaluated for specific gravity, pH, and sediment.

The observation period was 100 days after the operation, and individuals surviving 100 days or more were sacrificed to remove the transplanted kidney. In addition, individuals with postoperatively elevated creatinine levels, anuria for 6 days or more, and exacerbated clinical symptoms (frequent vomiting, diarrhea, inability to stand, loss of appetite) were sacrificed. The method of sacrifice was the same method as for anesthesia during surgery, followed by rapid administration of pentobarbital (100 mg/kg, IV) and potassium chloride (25 ml/head, IV). Ten minutes later, we confirmed cardiopulmonary arrest. At the time of sacrifice, blood was collected and ultrasonography and contrast-enhanced computed tomography (CT) were performed to evaluate the kidneys.

### Ultrasonography and Contrast-Enhanced CT Examination

Ultrasonography was performed under sedation to confirm blood flow and for morphological evaluation of the transplanted kidney. The pig was placed in the supine position. The vascular resistance coefficient (RI) was measured by the calculated systolic and diastolic blood flow rates using the pulsed Doppler method. RI is known to be high in rejection and kidney artery stenosis and has been shown to be associated with antibody-related rejection and acute tubular necrosis (40). In order to avoid variations in results due to differences in flow velocity depending on the measurement position, RI measurements were performed uniformly on the interlobar artery in all individuals. The RI was measured three times consecutively, and the average value was calculated. In contrast-enhanced CT examination, blood flow to the renal arteries and veins under sedation was observed. The pig was in the supine position, and iohexol (1 ml/kg) was rapidly administered intravenously for imaging.

### Histopathological Examination

The removed kidney, renal artery and vein, and ureter were immersed in 4% paraformaldehyde-phosphate buffer (4% PFA) and fixed at 4°C for 24–48 h. The kidneys were sectioned longitudinally to include the papilla centering on the cortex. The artery, vein, and ureter were divided along the lumen to a longitudinal section and were embedded. After embedding, thin-layer slices (3 µm) were prepared using a conventional method. Thin sections were stained with hematoxylin and eosin, Masson’s trichrome stain, and Elastica van Gieson stain. Histological examination was performed by blinded pathologists, and kidney graft pathology was assessed according to the Banff classification (41, 42).

## Results

### Recipient Information and Survival Days

**Table 1** shows the survival days, preservation time, and warm ischemic time in all groups. The warm ischemic time was the time from the placement of the transplanted kidney in the recipient to the reperfusion. Ischemia was slightly prolonged in the three individuals of H-DCD20 due to blood vessel site injury and bleeding. DCD0 was able to survive for 100 days. The day after the operation, appetite improved and urination was observed. Three H-DCD20 survivors (3/4 heads) survived for 100 days, whereas the remaining survivor had a nephrostomy tube under anesthesia 28 days after surgery due to severe ureteral obstruction. The general condition improved and was maintained. The surviving animals gradually recovered their appetite from postoperative days 2–3, and urinary excretion and normal feces were observed. NH-DCD20 was sacrificed 3–7 days after surgery due to exacerbation of clinical symptoms including anuria, vomiting, and deterioration of kidney function. Taken together, these findings suggest H-DCD20 enabled long-term survival despite of the more severe ischemic conditions than NH-DCD20.

**Table 1:** Information of groups.

	H-DCD 20 (n = 4)				NH-DCD 20 (n = 3)			DCD 0 (n = 1)
	No. 1	No. 2	No. 3	No. 4	No. 5	No. 6	No. 7	No. 8
Survival days	1	100	100	100	3	6	7	100
Warm ischemic time (min)	60	79	64	69	60	60	60	60
Preservation time (min)	240	60	240	240	60	60	60	17

### Blood and Urinary Tests

**Figure 2:**
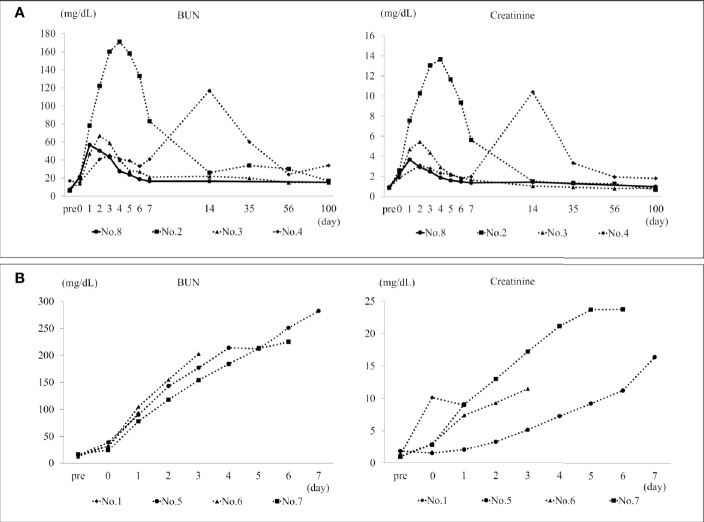
BUN and creatinine in the survival span. **(A)** The transition of BUN and creatinine in H-DCD20 individuals who were able to survive up to 100 days after surgery. The solid line shows DCD0. No. 4 has a temporary increase in BUN and creatinine due to ureteral obstruction. In other individuals, BUN and creatinine peaks within 5 days after surgery, and kidney function is stable until 100 days. **(B)** The transition of BUN and creatinine in individuals who died early after surgery. These continue to rise after the operation, and no improvement is observed. BUN, blood urea nitrogen. *H-DCD20: n=4, NH-DCD20: n=3, DCD0: n=1.

The BUN and creatinine of Nos. 2, 3, and 8 peaked on postoperative days 2–4 and gradually decreased; following that, the value remained stable (**Figure 2A**). No. 4 showed increased BUN and creatinine due to ureteral obstruction; however, BUN and creatinine improved with percutaneous nephrostomy tube placement and medical management under anesthesia. The BUN and creatinine of non-surviving individuals in H-DCD20 and all the individuals in NH-DCD20 continued to increase and did not improve (**Figure 2B**). Syndecan-1 levels are displayed in **Table 2**. The concentration in NH-DCD20 was higher than in DCD0 and H-DCD20 before and after surgery. Only the H-DCD20 (3/4) and DCD0 were able to provide urine after surgery. One individual in H-DCD20 and all the individuals in NH-DCD20 did show urine in the bladder by ultrasonography; however, urine could not be collected. The result of urinary testing 2 weeks after surgery showed that H-DCD20 (1.016 ± 0.004) had a lower specific gravity than DCD0 (1.029). In summary, the renal function in H-DCD20 could be earlier recovered than that of NH-DCD20 and remain stable.

**Table 2:** Concentration of Syndecan-1.

No.	H-DCD 20 (n = 2)		NH-DCD 20(n = 2)		DCD 0(n = 1)
	3	4	5	6	8
Pre (μg/ml)	5.08	2.48	10.32	10.99	5.24
Post (μg/ml)	9.06	5.46	16.46	15.13	7.34
Rate of variability (%)	40.0	78.3	120.3	59.4	37.7

ND, under than the limited value of measurement.

### Imaging Findings

**Figure 3:**
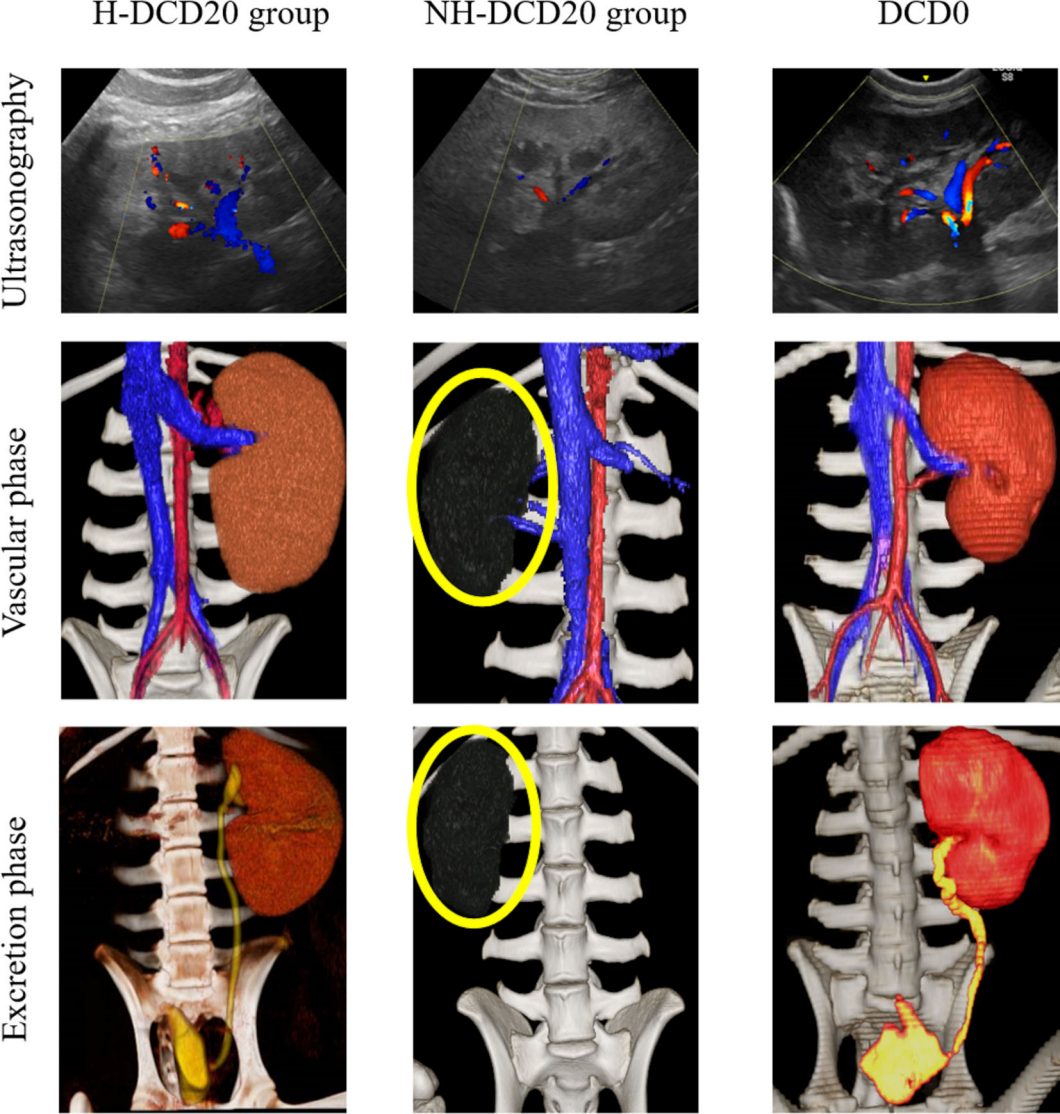
Ultrasonographic and contrast CT images. Upper row: Ultrasound images on the first day after the operation are shown. Renal blood flow in H-DCD20 individuals is clearly confirmed; however, some areas are difficult to see with a Doppler. Almost no renal blood flow is observed in NH-DCD20 individuals, and the renal parenchyma shows a hypoechoic image similar to that of surrounding tissues. Kidney blood flow in DCD0 is clearly confirmed, and branching of interlobar arteries and veins is observed from segmental arteries and veins. Middle/lower row: contrast-enhanced CT is used to demonstrate the vascular and excretory phases, and 3D stereoscopic images are shown. In H-DCD20 and DCD0, inflow of contrast medium into the kidney is observed, and the ureter and bladder are imaged. However, in individuals of NH-DCD20 and H-DCD20 without blood flow, the renal arteries are not imaged, and the ureter and bladder are not imaged during the excretory phase (yellow circle, transplanted kidney). CT, computed tomography; 3D, three-dimensional. *H-DCD20, n = 4; NH-DCD20, n = 3; DCD0, n = 1.

Ultrasonography showed clear blood flow in the kidneys of the segmental arteries in the surviving DCD0 and H-DCD20 individuals from postoperative day 1 to follow-up (**Figure 3**). One individual in the NH-DCD20 and H-DCD20 who died less than 7 days after transplantation had little or no kidney blood flow from the early postoperative period. In addition, the entire kidney was hypoechoic, making anatomical evaluation of the kidney difficult (**Figure 3**). The measured RI was higher for H-DCD20 than for DCD0 after surgery; however, it gradually decreased and became stable (**Table 3**). Only No. 5 could be measured in the NH-DCD20, and RI was higher than that of the other groups from the first day after surgery. All surviving individuals had clear contrast-enhanced images within the renal parenchyma using contrast-enhanced CT examination on day 100 (**Figure 3**). The renal arteries and veins were clearly visualized. The individuals who died within 7 days after operation did not show contrast medium influx into the renal parenchyma. Collectively, data indicate that clear blood flow in the surviving H-DCD20 individuals could be observed by ultrasonography and contrast-enhanced CT examination from postoperative day 1 to follow-up.

**Table 3:** RI value by ultrasonography.

		Day after operation							
		Day 1	Day 3	Day 5	1 week	2 weeks	1 month	2 months	Day 100
H-DCD20 (n = 4)	No. 1	NT	Dead	–	–	–	–	–	–
	No. 2	0.61	0.68	0.72	0.53	0.54	0.51	0.55	0.55
	No. 3	0.63	0.59	0.64	0.59	0.51	0.72	0.60	0.46
	No. 4	NT	NT	0.64	0.64	NT	0.63	0.75	0.71
NH-DCD20 (n = 3)	No. 5	0.71	0.78	0.63	0.82	Dead	–	–	–
	No. 6	NT	NT	Dead	–	–	–	–	–
	No. 7	NT	NT	NT	Dead	–	–	–	–
DCD0 (n = 1)	No. 8	0.50	0.51	0.53	NT	0.47	NT	0.51	0.66

NT, not tested (no blood flow or difficult to measure).

### Pathology Anatomy

No renal arteriovenous thrombus was observed in any individual who survived for 100 days (**Table 4**). In addition, individuals who survived for 100 days with H-DCD20 had ureteral anastomosis stenosis or adhesion to surrounding tissues, as well as dilated renal pelvis and ureters. The individuals who died within 1 week after surgery had thrombus in the kidney arteries (**Table 4**). In individuals whose blood flow could not be confirmed before dissection using ultrasound images, thrombus was confirmed in the renal arteries and in some of the segmental arteries and veins. In summary, individuals in H-DCD20 had no thrombus formation in the renal artery and vein; however, the individuals in NH-DCD20 had thrombus in the kidney arteries.

**Table 4:** Ultrasonographic and anatomical findings in the last day.

		H-DCD20 (n = 4)	NH-DCD20 (n = 3)	DCD 0 (n = 1)
Ultrasonographic findings	Renal artery flow	3/4	3/3	1/1
	Renal venous flow	3/4	3/3	1/1
	Ureteral dilation	3/4	0/3	0/1
	Pelvis dilation	3/4	0/3	0/1
Anatomical findings	Arterial thrombus	1/4	1/3	0/1
	Venous thrombus	1/4	0/3	0/1
	Ureteral dilation	3/4	0/3	0/1
	Pelvis dilation	3/4	0/3	0/1

### Histopathological Findings

**Figure 4:**
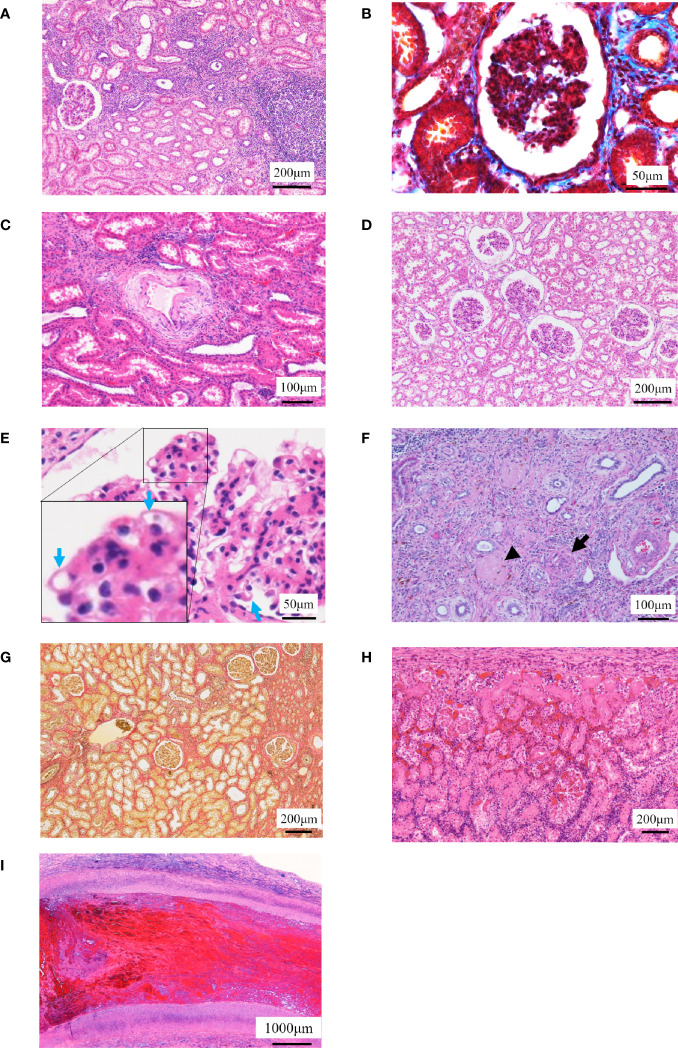
Histopathological examinations. **(A–C)** The histopathological images in No. 2 are shown. **(A)** Localized cell infiltration is observed, and cell infiltration is observed around the epithelial edema. **(B)** Mild fibrosis is observed (Masson’s trichrome staining). **(C)** Stenosis of the lumen in the kidney artery, necrosis of the intima and edema of the media, and cell infiltration in the adventitia are observed. **(D, E)** The histopathological images in No. 3 are shown. **(D)** Borderline change is observed to the extent that localized cell infiltration is observed. **(E)** Chronic glomerulosis due to thickening of the vascular loop (blue arrow) is shown with an increase in mesangial substrate. **(F, G)** The histopathological images in No. 4 are shown. **(F)** Arterial infarction (arrow) and venous infarction (arrowhead) are observed. **(G)** Fibrosis was mild but more extensive than No. 2 (Elastica van Gieson staining). **(H, I)** Representative histopathological images of individuals who died early after operation are shown. **(H)** Tubular necrosis is observed throughout the whole kidney. Infiltration of mononucleosis is observed near the capsule. **(I)** Thrombus formation is observed in the renal artery.

The histopathological images are shown in **Figure 4**. DCD0 (No. 8) and the surviving individuals (Nos. 2, 3, and 4) showed chronic tissue changes. The kidney of No. 2 showed moderate tubular inflammation and glomerulitis and mild mononuclear cell infiltration into the stroma. Histological images showed mild chronic allograft nephropathy such as tubular atrophy and interstitial fibrosis. Stenosis of the arterial lumen, necrosis of the intima, edema of the media, and cell infiltration into the adventitia were observed (**Figures 4A–C**). These were comprehensively evaluated and found to be type I chronic allograft nephropathy, according to the Banff classification (**Table 5**). In No. 3, the glomerulitis was moderate; however, there was no cell infiltration into the stroma. Additionally, the tubular atrophy and tubular inflammation were mild (**Figures 4D, E**). Therefore, No. 3 was identified to have no chronic allograft nephropathy. In No. 4, mild interstitial fibrosis and atrophy of renal tubules were observed, and cellular infiltration, arterial intima, and venous infarction were noted in some areas (**Figures 4F, G**). Thus, we concluded that this was a type I chronic rejection, according to the Banff classification (**Table 5**). Three of the four individuals who died within 7 days showed severe tubular necrosis over the entire area, and cellular proliferation and hemorrhage from the intima to the adventitia of the renal arteries and veins were detected (**Figure 4H**). In Nos. 1 and 7, there were infarcts due to thrombus in the renal arteries (**Figure 4I**).

**Table 5:** Histopathological findings for kidney allograft.

No.	H-DCD 20 (n = 4)				NH-DCD 20 (n = 3)			DCD 0 (n = 1)
	1	2	3	4	5	6	7	8
Glomerulitis	/	2	2	2	3	/	/	2
Interstitial mononuclear cell infiltration	0	1	0	1	3	/	/	0
Tubulitis	0	2	1	3	3	1	2	0
Intimal arteritis	0	1	0	1	1	0	0	0
Chronic allograft nephropathy type/grade	0	**I**	0	**I**	**I**	0	0	0
Chronic transplant glomerulopathy	/	3	1	3	2	/	/	0
Interstitial fibrosis	0	1	0	1	2	0	0	0
Tubular atrophy	0	1	1	1	3	0	0	1
Mesangial matrix	/	3	3	3	3	/	/	1
Vascular fibrous intimal thickening	0	1	1	2	0	0	0	0

Grade 0, no change; 1, mild; 2, moderate; 3, severe;/, no examination.

Chronic allograft nephropathy type/grade.

I. Mild interstitial fibrosis and tubular atrophy (<25% of cortical area).

II. Moderate interstitial fibrosis and tubular atrophy (26%–50% of cortical area).

III. Severe interstitial fibrosis and tubular atrophy/loss (>50% of cortical area).

The supplementary table shows the number of drops in the infusion line when rinsing with ETK. As in previous reports, H-ETK (n = 3) has a higher number of drops per minute than NH-ETK (n = 3). The supplementary figure is the result of histopathological examination. This result was examined after the graft was treated by the same procedure as in this study and immediately fixed. As a result, the hydrogen-treated graft (hydrogen; n = 1) tended to have a mild tubular degeneration to necrosis with dilatation score compared to the non-hydrogenized graft (control; n = 1).

Collectively, data indicate that the surviving individuals in H-DCD20 showed no to mild chronic allograft nephropathy since there was a possibility that the hydrogen improved an acute tubular degeneration and necrosis compared to the non-hydrogen-treated kidney.

## Discussion

To the best of our knowledge, this is the first study to observe long-term prognosis after transplantation using hydrogen in the ischemic kidney of mature minipigs and to inhibit chronic rejection response using a strict multi-immunosuppressant protocol. 　

NH-DCD20 showed severe kidney damage in the acute phase and was sacrificed, whereas H-DCD20 quickly recovered from IRI in the acute phase and all survived for long periods except for one individual. Kidney transplant dysfunction can lead to 6-day survival. Therefore, we believe that the cause of an early death of the individual in H-DCD20 was not transplanted kidney dysfunction; rather, there were other causative factors. We speculate that hydrogen improved microcirculation and that this was one of the factors that caused the difference in the long-term survival between the two groups.

In human studies, kidney allograft thrombosis accounts for 2%–7% of graft loss (43). The causes include technical factors, vascular endothelial damage, decreased cardiac output, acute tubular necrosis, and acute rejection. In the case of ischemic organ transplantation, rejection is strongly associated with microcirculatory changes induced by inflammatory cytokines due to IRI (44, 45). Hydrogen has been shown to suppress the production of the major inflammatory cytokines IL-1β, IL-6, and TNF-α, and it has anti-inflammatory effects that reduce tissue damage (29, 34). Saito et al. reported that using hydrogenated preservatives for lung transplantation reduced the expression of IL-1β and 8-hydroxyguanosine and reduced the number of neutrophils attached to the vascular wall and hydrogen-inhibited vascular endothelial damage (29). In addition, ETK potentially contributes to improving microcirculation. In actual human clinical cases, its clinical application in lung transplantation has been reported (46). Although its application in kidney transplantation as a clinical example has not been reported, in experimental animals, we has previously shown prolongation of individual survival by ETK preservation in ischemic kidney transplant rat models compared to the University of Wisconsin solution and lactated Ringer’s solution (37). The high-sodium/low-potassium composition of ETK and the trehalose contained in it are thought to suppress vasoconstriction due to high potassium concentration and cell edema, reduce reperfusion injury, and inhibit further ischemia. Chronic tissue changes can be expected to suppress inflammatory cytokines and reactive oxygen species and improve ischemia. Therefore, the combination of H and ETK may be one of the leading protocols for ischemic kidney transplantation.

The glycocalyx present in vascular endothelial cells is an extremely fragile structure, consisting of proteoglycan, glycoprotein, and glycan; it is responsible for maintaining microcirculation and barrier function of blood vessels and easily disintegrates upon stimuli such as IRI and cytokines (7, 34, 47, 48). The glycocalyx is present on the surface of glomerular capillaries and podocytes in the kidney and has been shown to be exfoliated by exposure to lipopolysaccharides. Hydrogen is thought to protect the glycocalyx (49). There were decreased expression levels of syndecan-1 in the glycocalyx when hydrogen was inhaled in a non-traumatic hemorrhagic shock rat model, showing that hydrogen contributed to the prolongation of survival (49). One study showed that an increase in syndecan-1 levels in tubular epithelial cells was associated with improved graft function and prolonged engraftment (50). Furthermore, syndecan-1 knockout mice were often affected by renal IRI and were prone to fibrosis. Although measured syndecan-1 levels increased postoperatively in all groups, the fluctuating rate was lower in NH-DCD 20 than in H-DCD 20. The reason may be as follows: NH-DCD 20 did not effectively rinse out cytokines and oxidants, thereby inducing thrombus formation immediately after reperfusion. Systemic circulation of syndecan-1 did not occur during reperfusion. This can further be determined by the appearance of the kidney after reperfusion (**Figures 2B, C**). This suggests that the increasing serum levels of syndecan-1 may have been underestimated. In contrast, in H-DCD 20, the rate of change of syndecan-1 expression was relatively high because hydrogen removed microthrombi and blood entered the systemic circulation from the graft.

No chronic rejection was observed in the transplanted kidney with long-term engraftment of H-DCD 20, and there were individuals with the same histopathological image as DCD 0. In previous studies, it has been shown that ischemic kidney transplantation is likely to induce chronic rejection. The ischemic state of the transplanted kidney contributes to non-immune chronic rejection, and the induction of acute inflammation and rejection by IRI results in chronic organic changes in tissue, including thickening of the arterial wall. We evaluated the rate of decrease during ETK flow within the kidney and confirmed that rapid flow of ETK decreased in the line, similar to previous reports (**Supplementary Table**). Furthermore, we confirmed that the histopathological findings of the kidney immediately after rinsing with ETK showed no difference; however, tubule degeneration associated with dilation was mild in the kidney using ETK containing hydrogen (**Supplemental Figure**). Currently, most studies on chronic rejection have been using rats with low-responder combinations, and immunosuppressants have often been used alone for short periods of time. In the present study, we did not confirm the haplotypes of SLA. Most MHC haplotypes of the microminiature pigs used have been identified; however, this is currently under study (36). SLA has multiple haplotypes, as in humans. This suggests the requirement for multi-immunosuppressant protocols similar to actual clinical practice. Furthermore, recently, a mechanism called IL-6 amplifier has been considered for MHC class II-related inflammatory reactions. This indicates that CD4+ T cell-derived cytokines such as IL-17A may lead to sustained activation of IL-6 due to T-cell activation and local accumulation that are not related to antigen specificity (51). To support this, it is shown that synaptotagmin-17, a type of urinary exosome, is increased in association with IL-6 amplifiers in patients with chronic active antibody-mediated rejection in kidney transplantation (52). These were considered that may be relating the suppression of cytokines by hydrogen and may affect the results of mild fibrosis and tubular atrophy in the H-DCD20 (29, 34).

Hydroxyl radical (OH^−^) and reactive oxygen species cause oxidative damage to kidney tissue, producing cytokines such as TGF-β1 and tubular cells, causing epithelial-mesenchymal transition (EMT). EMT causes fibrosis by filling the tubular interstitial with fibrotic substances such as vimentin and α-smooth muscle actin, causing arterial ischemia and atrophy of glomeruli and kidney tubules. Therefore, it was suggested that the hydrogen-dissolved organ preservation solution suppresses the upper cascade of fibrosis by removing hydroxyl radicals, reactive oxygen species, and inflammatory cytokines, and may contribute to the reduction of chronic rejection.

The ischemic time affected long-term survival in the ischemic kidney transplant model of miniature pigs used in this study. In humans, it has been shown that the retention and survival rate of transplanted organs decreased when the ischemic time was 30 min or more (53), and the ischemic time compatible with survival after transplantation differs depending on the animal species. Previous studies in miniature pigs showed ischemic times of 30 min and observation periods of less than 1 week (26). In addition, we noted that survival after kidney transplantation in miniature pigs with an ischemia time of 30 min was difficult for all (n = 9, unpublished data). Autologous transplantation using general domestic pigs and miniature pigs involve young pigs and there is no use of immunosuppressants; hence, the ischemic time is 30–60 min (54, 55). Therefore, it was speculated that the maturity of microminiature pigs was one of the factors giving rise to the difference in viability despite using the same animal species. As mentioned previously, pigs generally used in transplantation experiments are young, typically less than 6 months old. It cannot be denied that this acts as a kidney protective effect because many factors such as immune response and growth hormone can be confounded during the growth period (56), and this precludes extrapolation to humans. The ischemic kidney transplantation model of mature miniature pigs created in this study is expected to contribute significantly to preclinical studies in humans.

The limitations of this study were the small number of individuals included and the limited data obtained, unknown detail SLA types, and the lack of complete elucidation of the mechanism by which the improvement tendency of chronic rejection occurred. Regarding this point, we are considering experiments considering the combination of SLAs in the future. The purpose of this study was to confirm long-term survival and the accompanying changes in chronic tissue response; IHC, PCR, ELISA, and flow cytometry were not performed on tissue sections. However, Abe et al. reported that dissolving hydrogen in the UW organ preservation solution during renal transplantation in rats suppressed tubular apoptosis and reduced interstitial macrophage infiltration (57). We think this can be observed in miniature pigs as well. In addition, we plan to analyze the inflammatory cytokine cascade and its associated cell-mediated immune response. Nevertheless, it is clear that hydrogen improves the blood flow environment in the ischemic kidney in ischemic kidney transplantation in mature pigs. Hydrogen enables rapid recovery of kidney function and long-term survival in the context of administration of multidrug immunosuppressive therapy. Furthermore, the fact that chronic rejection was not observed or was mild in ischemic kidney transplantation of pigs, we can infer that this was mediated by hydrogen. This factor can contribute significantly in explaining the chronic rejection mechanism.

In conclusion, hydrogen induced a positive effect on IRI, possibly suppressing thrombus formation and microcirculation for lethal ischemic kidney transplantation in miniature pigs. Furthermore, findings suggested suppression of chronic rejection in long-term engrafted kidney tissue and showed a relationship between inflammation and rejection in the acute phase and chronic tissue changes. This is a significant preclinical model, and we believe that this model will provide further insight into the mechanism of chronic rejection.

## Data Availability Statement

The original contributions presented in the study are included in the article/**Supplementary Material**. Further inquiries can be directed to the corresponding author.

## Ethics Statement

The animal study was reviewed and approved by The Research Council and Animal Care and Use Committee of Kitasato University (approval number: 18-133, 19-086).

## Author Contributions

KN, SI, and EK contributed substantially to the conception and design of the work. SI made the same contribution as KN in this research. KN, SI, KT, SO, MS, and EK contributed to the acquisition, statistics, analysis, and interpretation of data. EK supervised and confirmed all the data and supervised the opinions of all the co-authors. All co-authors reviewed the final version of this paper and agreed to submit it. The authors agreed to be accountable for all aspects of the work in ensuring that questions related to the accuracy or integrity of any part of the work are appropriately investigated and resolved. All authors contributed to the article and approved the submitted version.

## Funding

This work was supported by grants from Doctors Man Co., who had no role in study design, data collection and analysis, decision to publish, or preparation of the manuscript (Grant number: 2225-7257, 2225-7265).

## Conflict of Interest

Co-authors of this manuscript, EK and MS, are medical advisors to Doctors Man Co., Ltd.

The remaining authors declare that the research was conducted in the absence of any commercial or financial relationships that could be construed as a potential conflict of interest.
