# Supplementary material for: Prevention of Chronic Rejection of Marginal Kidney Graft by Using a Hydrogen Gas-Containing Preservation Solution and Adequate Immunosuppression in a Miniature Pig Model
Source: Front Immunol. 2021 Feb 17;11:626295. doi: 10.3389/fimmu.2020.626295 (PMC7925892; doi:10.3389/fimmu.2020.626295)
Supplement: Supplementary file 1 [file DataSheet_1.docx]

Supplementary Figure. Tubular degeneration to necrosis with dilatation

The percentage of the area affected was estimated for the number of tubular degenerations was scored as follows: 0 = 0-5%, 1 = 5-10%, 2 = 11~25%, 3 = 26~45%,

4 = 46-75%, 5= >76%
