# Supplementary material for: Prevention of Chronic Rejection of Marginal Kidney Graft by Using a Hydrogen Gas-Containing Preservation Solution and Adequate Immunosuppression in a Miniature Pig Model
Source: Front Immunol. 2021 Feb 17;11:626295. doi: 10.3389/fimmu.2020.626295 (PMC7925892; doi:10.3389/fimmu.2020.626295)
Supplement: Supplementary file 2 [file Table_1.docx]

| (drops/min) | H-ETK (n=3) | NH-ETK (n=3) |
| --- | --- | --- |
| A | 210 | 90 |
| B | 168 | 88 |
| C | 89 | 73 |
| Average | 155.7 | 83.7 |
| SD | 50.2 | 7.6 |

Supplementary Table. Drop rate by the ETK rinse to removal kidney

The table was indicated how many rates did the ETK drop in the line from catheter inserted kidney artery. The H-ETK was more quickly flown than NH-ETK in kidney.
